# Economic and feasibility comparison of the dRIT and DFA for decentralized rabies diagnosis in resource-limited settings: The use of Nigerian dog meat markets as a case study

**DOI:** 10.1371/journal.pntd.0008088

**Published:** 2020-02-28

**Authors:** Eze U. Ukamaka, Andre Coetzer, Terence P. Scott, Boniface M. Anene, Romanus C. Ezeokonkwo, Chika I. Nwosuh, Louis H. Nel, Claude T. Sabeta

**Affiliations:** 1 Department of Veterinary Medicine, University of Nigeria, Nsukka, Nigeria; 2 Department of Biochemistry, Genetics and Microbiology,University of Pretoria, Pretoria, South Africa; 3 Global Alliance for Rabies Control SA NPC, Pretoria, South Africa; 4 Department of Veterinary Parasitology and Entomology, Faculty of Veterinary Medicine, University of Nigeria, Nsukka, Nigeria; 5 Rabies Unit, Central Diagnostic Laboratory, National Veterinary Research Institute, Vom, Jos, Nigeria; 6 OIE Rabies Reference Laboratory, Agricultural Research Council-Onderstepoort Veterinary Institute, Pretoria, South Africa; 7 Department of Veterinary Tropical Diseases, University of Pretoria, Pretoria, South Africa; Centers for Disease Control and Prevention, UNITED STATES

## Abstract

**Background:**

Rabies lyssavirus (RABV) is the aetiologic agent of rabies, a disease that is severely underreported in Nigeria as well as elsewhere in Africa and Asia. Despite the role that rabies diagnosis plays towards elucidating the true burden of the disease, Nigeria–a country of 180 million inhabitants–has a limited number of diagnostic facilities. In this study, we sought to investigate two of the World Organization for Animal Health (OIE)-recommended diagnostic assays for rabies–viz; the direct fluorescent antibody test (DFA) and the direct rapid immunohistochemical test (dRIT) in terms of their relative suitability in resource-limited settings. Our primary considerations were (1) the financial feasibility for implementation and (2) the diagnostic efficacy. As a case study, we used suspect rabies samples from dog meat markets in Nigeria.

**Methods/Principal findings:**

By developing a simple simulation framework, we suggested that the assay with the lowest cost to implement and routinely use was the dRIT assay. The costs associated with the dRIT were lower in all simulated scenarios, irrespective of the number of samples tested per year. In addition to the cost analysis, the diagnostic efficacies of the two assays were evaluated. To do this, a cohort of DFA-positive and -negative samples collected from dog meat markets in Nigeria were initially diagnosed using the DFA in Nigeria and subsequently sent to South Africa for diagnostic confirmation. In South Africa, all the specimens were re-tested with the DFA, the dRIT and a quantitative real-time polymerase chain reaction (qRT-PCR). In our investigation, discrepancies were observed between the three diagnostic assays; with the incongruent results being resolved by means of confirmatory testing using the heminested reverse transcription polymerase reaction and sequencing to confirm that they were not contamination.

**Conclusions/Significance:**

The data obtained from this study suggested that the dRIT was not only an effective diagnostic assay that could be used to routinely diagnose rabies, but that the assay was also the most cost-effective option among all of the OIE recommended methods. In addition, the results of our investigation confirmed that some of the dogs slaughtered in dog markets were rabies-positive and that the markets posed a potential public health threat. Lastly, our data showed that the DFA, although regarded as the gold standard test for rabies, has some limitations—particularly at low antigen levels. Based on the results reported here and the current challenges faced in Nigeria, we believe that the dRIT assay would be the most suitable laboratory test for decentralized or confirmatory rabies diagnosis in Nigeria, given its relative speed, accuracy, cost and ease of use.

## Author summary

The under-reporting of rabies in Nigeria may partly be attributed to a lack of adequate diagnostic facilities, which in turn leads to its neglect. The role that dog meat markets play in the epidemiology of rabies in Nigeria remains poorly defined as the activities in these markets are neither properly regulated nor reported to appropriate veterinary and public health authorities. Several diagnostic assays have recently been recommended for rabies diagnosis by the OIE. However, challenges faced in resource-limited countries like Nigeria make the use of some of the recommended assays challenging or impractical. During this study, we evaluated the most feasible OIE-recommended diagnostic assay (based on both diagnostic efficacy and cost) for decentralized laboratory diagnosis of rabies in Nigeria using dog meat markets as a case study. Our findings suggest that the dRIT is best suited for implementation in Nigeria. In addition, our results highlight the importance of confirmatory testing at a central veterinary laboratory, ensuring accurate diagnosis to facilitate improved surveillance for the progression of Nigeria towards eliminating dog rabies.

## Introduction

The *Lyssavirus* genus (family: *Rhabdoviridae*, order: *Mononegavirales*) consist of 16 viral species that are all capable of causing the disease rabies an invariably fatal zoonosis typified by an encephalomyelitis in mammalian species [1, 2]. In contrast to the 15 rabies-related lyssaviruses that appear to have specific geographical distributions, the prototype species rabies lyssavirus (RABV) is responsible for the death of an estimated 59,000 humans in the developing countries of Africa and Asia annually [3]. Of these, 21,000 (36%) occur in Africa where rabies has the highest per-capita death rate [3]. In Nigeria alone, an estimated 1,600 humans die of rabies annually, the third highest number of rabies deaths for Africa a continent consisting of 54 countries [3]. Furthermore, since dogs are the principal reservoir species of RABV and not all rabies-suspect cases are submitted to diagnostic laboratories the number of rabies cases in this species is likely to be several magnitudes higher, highlighting the extent of the animal and public health challenges posed by rabies [4]. These challenges are, however, avoidable because rabies is a preventable disease through either pre- or post-exposure prophylaxis (PrEP or PEP) in humans and mass vaccination of dog populations. Considering that the average daily income in Nigeria is about US$1–2 per person and that the average cost of human rabies vaccine in Africa is US$40 per dose [5] (and slightly more (US$42 per dose) in Nigeria) PEP is understandably unaffordable for most people that are at-risk in the country. In addition, the use of PEP is a reactive rather than preventative measure and does not provide a long-term solution to the control and eventual elimination of canine rabies. Indeed, the mass vaccination of dog populations is the only cost effective and feasible approach to this end [6].

Despite rabies control and elimination being economically and practically feasible, the inadequate control of rabies in developing countries can most often be attributed to the cycle of neglect. This phenomenon explains the under estimation or non-recognition of the burden of rabies, resulting in low governmental prioritization and a general lack of political will to control and eliminate the disease [7]. Consequently, limited resources are invested towards understanding and controlling the disease, further aggravating current challenges. This is exemplified in Nigeria, particularly in the rural areas where large, unvaccinated dog populations are found in both rural communal settlements and dog meat markets. While the exact number of dog meat markets are not known, previous studies have shown that they can contribute significantly to the burden of rabies in Nigeria [8, 9]. Although the slaughter and processing of dogs for human consumption is a source of livelihood for many people in Nigeria [10,11, 12, 13], previous findings demonstrated that rabies is prevalent in dogs slaughtered in these markets, posing a major public health risk [8, 11, 12, 14, 15, 16, 17,18,19, 20, 21]. Despite the fact that a number of research papers (*n = 11)* have reported the possible public health impact of rabies in dog meat markets in Nigeria [8, 11, 12, 14, 15, 16, 17,18,19, 20, 21], active surveillance programs are limited and, in turn, result in an unquantified risk for the butchers, merchants and animal handlers.

The DFA test is the gold standard diagnostic assay for rabies. It is not only the World Organization for Animal Health’s (OIE) most widely implemented recommended diagnostic assays for rabies but also the only diagnostic assay routinely applied in Nigeria [22]. While the DFA test is ideal for developed countries, it has been shown to be less than ideal in developing countries given the costs involved in acquiring and maintaining a fluorescent microscope, the limited infrastructure, and the inadequate or non-existent quality control within the diagnostic laboratories [9, 22, 23, 24]. One assay that has shown great promise in terms of overcoming these challenges in developing countries is the direct, rapid immunohistochemical test (dRIT)–an effective diagnostic assay that detects the presence of lyssavirus antigen using a compound light microscope [25, 26, 27, 28, 29, 30, 31]. Indeed, the versatility and efficacy of the dRIT assay has recently resulted in this diagnostic assay being recommended by the OIE as a diagnostic method for rabies [22]. Furthermore, many claims have suggested that the dRIT is a more cost-effective diagnostic assay when compared with the DFA based on equipment requirements, but no clear cost analysis has been undertaken to substantiate these claims. Another diagnostic test recently recommended by the OIE for confirmatory rabies diagnosis is either a conventional polymerase chain reaction (PCR) or a quantitative real-time PCR (qRT-PCR) that has passed the OIE Standards for validation [22]. While the detection of amplified viral nucleic acid is a highly effective diagnostic principle, resource-limited countries often lack the necessary infrastructure–namely an appropriate cold chain and dedicated PCR rooms to minimize contamination during amplification of specific viral nucleic acids [32, 33]. These limitations prevent the implementation of advanced molecular techniques in Nigeria [9, 23]. Therefore, we hypothesize that the dRIT is presently the best-suited assay for the decentralized laboratory diagnosis of rabies in Nigeria.

In Nigeria, apart from the Central Diagnostic Division of National Veterinary Research Institute (NVRI) (Vom, Plateau State) which is the national reference center, there are only two centers located in the northern and western regions of the country that undertake rabies diagnosis. This has resulted in a lack of diagnostic support in the southern and eastern regions of the country where an increase in the number of dog meat markets has been observed due to the increasing popularity of dog meat [13]. Indeed, there is practically no rabies diagnosis being undertaken in South-East Nigeria given the lack of government subsidy allocated towards the shipment of specimens to diagnostic laboratories.This situation has resulted in the true burden of rabies in South-East Nigeria being grossly underestimated.

In an effort to advocate for improved surveillance in Nigeria, the aim of this study was to evaluate and compare the costs associated with the routine implementation of two OIE-recommended diagnostic assays (the DFA and the dRIT) in a resource-limited country’s existing diagnostic facility. In addition to the cost analysis, we endeavored to compare the diagnostic efficacy of the assays to provide a comprehensive overview of their applicability for decentralized diagnosis (or central diagnosis in other resource-limited settings). To this end, the diagnostic sensitivity and specificity of three assays (DFA, dRIT and a qRT-PCR (for diagnostic confirmation)) were determined using a cohort of samples collected from dog meat markets in the South-East region of Nigeria. The results provided an evaluation of the proficiency and applicability of the various diagnostic assays that could be considered for future decentralized rabies diagnosis in Nigeria while also providing an insight into the potential public health hazards of rabies in the selected dog meat markets.

## Materials and methods

### Ethical statement

This study was conducted in adherence to the Faculty of Veterinary Medicine, University of Nigeria, Nsukka's guidelines for animal husbandry which corresponds with the National Institute of Health (NIH) guidelines [34]. The brain specimens were collected with the consent of dog traders, dog meat sellers and pet owners (Approval number: UNN/eTC/14/68625).

The animal experimental protocols, animal caging and care, as well as end points for the animal experiments, were approved by the Animal Ethics Committee for the use of “mice and other living vertebrates for research, diagnostic procedures and product development” (ARC-OVI, South Africa; Approval number: P10000045).

The Animal Ethics Committee (AEC) of the University of Pretoria (South Africa) provided ethical approval for the implementation of the dRIT and qRT-PCR assays as described here (Approval numbers: EC027-16).

### Cost comparison analysis of DFA and dRIT implementation

To gain an improved understanding of the versatility of the two rabies diagnostic assays of interest (DFA and dRIT), the financial implications of implementation in a simulated resource-limited country were determined–an approach that, to the best of our knowledge, has only been inferred to-date. To this end, a simulation framework was developed using a modelled representation of a resource-limited country that has facilities containing the basic infrastructure (e.g. basic laboratory equipment, electricity, water, etc.). Furthermore, we relied on two data sets: i) laboratory throughput (based on three throughput scenarios, i.e. 50, 500 and 1000 samples per annum) and; ii) cost data (based on both capital investment and operational (fixed and variable) costs calculated over a one-, five- and ten-year period) to determine the cost per sample diagnosed.

#### Laboratory throughput

When considering laboratory throughput, the financial implications of diagnosing a low (*n = 50* samples per annum), medium (*n = 500* samples per annum) or high (*n = 1000* samples per annum) number of samples over a period of multiple years was considered (See S1 File for additional details).

#### Cost data

In our investigation we considered the capital investment and operational costs to obtain a clearer representation of the various financial components associated with each test as well as the impact on the price per diagnostic reaction.

The capital investment considered in our investigation consisted of all the costs that were directly associated with procuring the equipment required for each diagnostic assay, while also implementing a multi-year analysis to account for equipment investment as it was unlikely that a government would invest the money as a single year investment.

The operational cost was further split into fixed and variable costs. The fixed costs attributed to each of the two diagnostic assays in our investigation were the labour costs associated with a laboratory diagnostician, cost of the annual microscope service and the vaccination of the diagnostic technician. The variable costs included those associated with the diagnostic reagents and consumables that would be required to implement either of the two assays under investigation. Furthermore, the variable costs considered the direct influence of the number of samples subjected to a single diagnostic run. In addition, we calculated the total variable cost per annum by multiplying the calculated reagent cost per run with the theoretical number of samples diagnosed per year.

To determine the total cost per diagnostic assay for both the DFA and dRIT assays, we divided the final cost (consisting of both capital investment and operational costs) by the average number of samples tested per year (See S1 File. “Supporting document1” for additional details).

### Determining the diagnostic efficacy of three diagnostic assays that could be used for decentralized rabies diagnosis in Nigeria

#### Study location

**Fig 1:**
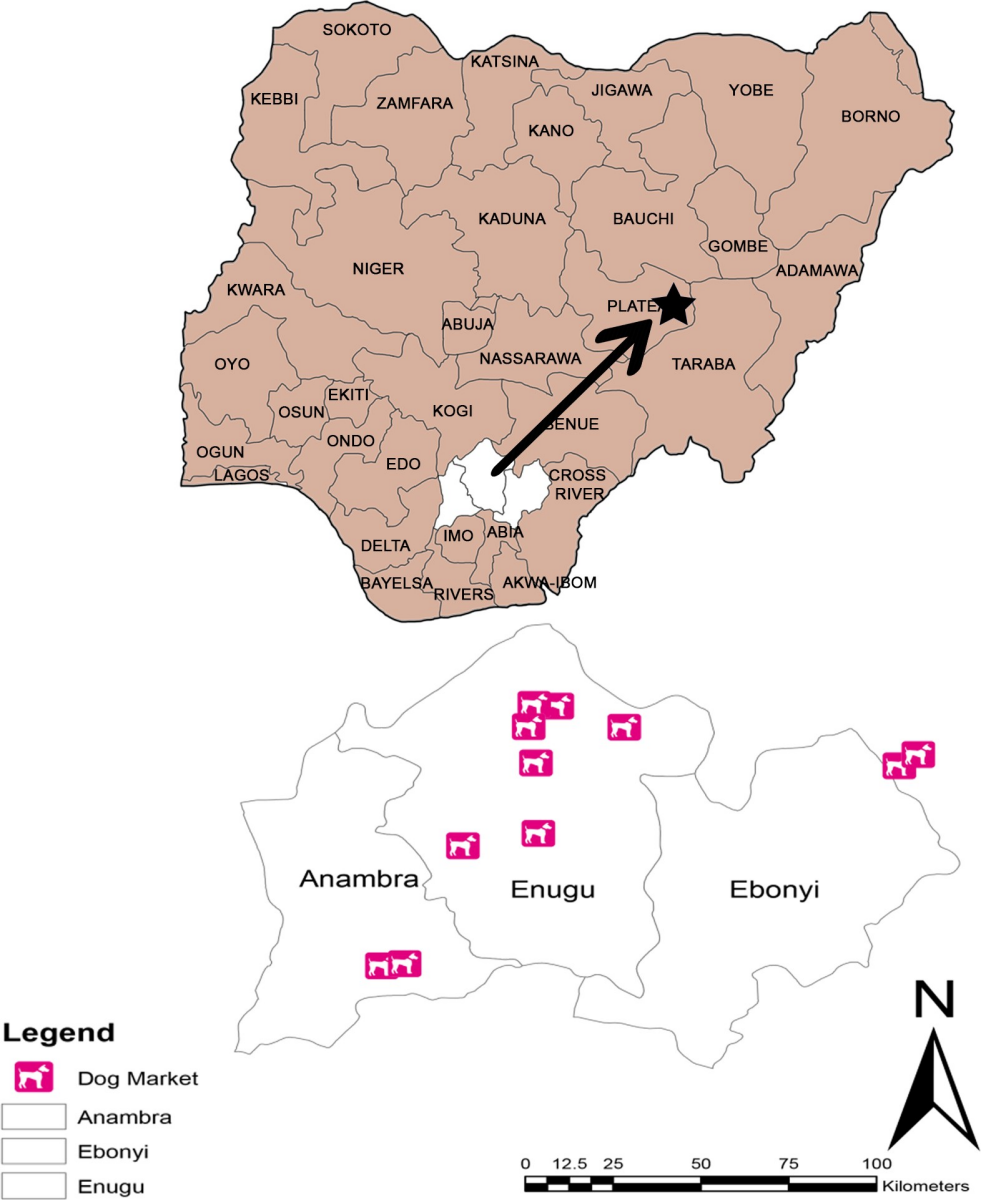
Map showing area of sample collection (Anambra, Enugu and Ebonyi State). Map created using ArcView 8.0 software.*Arrow showing the distance from South East region to the rabies diagnostic reference laboratory.

The samples included in this study originated from three selected states (Anambra, Ebonyi, and Enugu) in South-East Nigeria (Fig 1). These states, covering a total land area of 17,545 km^2^, are inhabited by approximately 16,381,729 people [35] and have approximately nine dog meat markets that serve the population.

#### Specimen collection

Specimens were collected between October 2015 and July 2016 after communications were sent to local animal health professionals, as well as the heads of dog markets and restaurant owners who engage in the dog meat business. All of the dog heads included in this study (*n = 278*) were collected in two batches during the investigation and were transported to the Department of Veterinary Pathology and Microbiology at the University of Nigeria, Nsukka.

The first batch (*n = 260*) consisted of brain tissue specimens collected randomly from dog markets. The second batch (*n = 18*) of samples were also from these markets, but were collected from rabies-suspect dogs (i.e. dogs that demonstrated behavioral changes such as restlessness, irritability, excitability and shyness or in some cases those that may have had injuries of unknown origin). All the samples were collected and stored frozen at -20°C until they were subjected to the DFA test in Nigeria.

#### Direct fluorescent antibody test

All the collected specimens (*n = 278*) were transported in appropriate cold storage (cooler box containing ice packs) to the Rabies Unit at NVRI in Plateau State, Nigeria. At NVRI, the DFA assay was undertaken on all the samples according to a standard operating procedure [36], with the diagnosticians treating homogenized tissue impression smears with a fluorescein-isothiocyanate (FITC)-labelled monoclonal antibody (MAb) preparation (Fujirebio, Japan) in order to confirm any false results.

**Fig 2:**
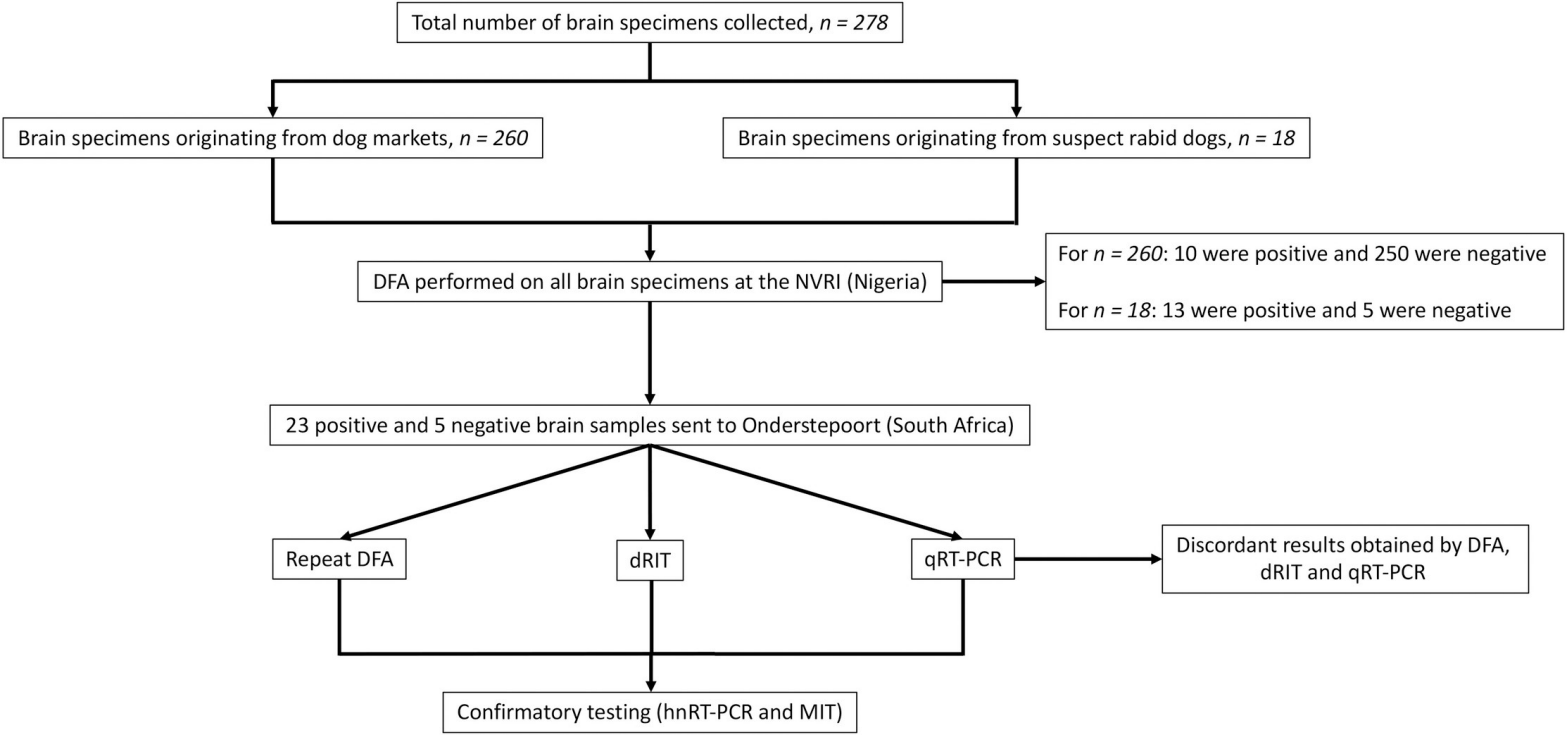
Flow diagram of specimens’ collection and diagnosis.

All brain tissues diagnosed as DFA-positive (*n = 23*), and five brain tissue samples collected from the suspect rabid animals that were diagnosed as rabies-negative in Nigeria, were shipped to the Agricultural Research Council-Onderstepoort Veterinary Institute (ARC-OVI), OIE Rabies Reference Laboratory (South Africa) for diagnostic confirmation (see flow diagram; Fig 2).

The 28 brain samples (23 DFA-positive and 5 DFA-negative collected from suspect rabid animals) were sent to the ARC-OVI Rabies Unit, and re-tested in a blinded protocol by an experienced diagnostician using the same DFA protocol as that used in Nigeria, with the exception of using a FITC-labelled anti-ribonucleoprotein polyclonal antibody (PAb) preparation (BIORAD) as opposed to the MAb preparation used in Nigeria (Fujirebio, Japan) [36].

#### Direct rapid immunohistochemicaltest diagnosis

The 28 brain samples were blindly tested using the dRIT assay as described previously [25]. An experienced diagnostician tested each sample by treating the homogenised tissue impression smears with a biotinylated anti-ribonucleoprotein PAb preparation (batch N4-15, ARC-OVI, Rabies Unit)–a PAb different to that used in the DFA at the ARC-OVI.

#### Molecular diagnosis

The total viral RNA of each of the 28 brain samples was Trizol-extracted according to the guidelines of the manufacturer (Sigma Aldrich, USA) and subjected to the OIE-validated “one-step” qRT-PCR assay, targeting the partial nucleoprotein gene of the lyssavirus genome [37]. The specific primer and probe set used in the reported protocol have been shown to have a lower limit of detection approaching ten RNA copies per reaction, while also being able to detect the majority of the viral species in the *Lyssavirus* genus (Table 1) [37]. All of the extracted RNAs were subsequently stored at -70°C for further analyses.

**Table 1:** Oligonucleotide primers and probe sequences for the “one-step” real-time qRT-PCR amplification of the nucleoprotein gene of the lyssavirus genome.

Primer or probe	Sequence (5’-3’)	Position on lyssavirus genome*
550B	GTRCTCCARTTAGCRCACAT	647–666
541lys	CACMGSNAAYTAYAARACNAA	541–561
620lyssa (probe)	FAM-CATCACACCTTGATGACAACTCACAA-BHQ-1	620–645

* Nucleotide positions are numbered according to the Pasteur Virus genome sequence (Genbank accession number: M13215)

#### Hemi-nested Reverse Transcription Polymerase Chain Reaction (hn-PCR) diagnosis

In an effort to further resolve any discrepant results, the viral RNA from the six samples with discrepant results between the three OIE-recommended assays (DFA, dRIT and qRT-PCR) were subjected to an established hn-PCR reaction [38]. At the conclusion of the reverse transcription reaction, the generated cDNA (*n = 6*) was subjected to amplification using the 001lys and 304 primer pair, followed by a hn-PCR reaction using the 001lys and 550B primer pair (Table 2)[39]. The amplified DNA products were visualized under UV transillumination after electrophoresis through 1% ethidium bromide stained agarose gels (Labnet, Power Station 300), with a 100 bp DNA ladder as the molecular weight marker (Promega, U.S.A). The PCR products of the five discrepant results were subjected to Sanger sequencing and the data analysis was conducted using Molecular Evolutionary Genetics Software Version 7 (MEGA7) [40]. The Kimura’s two parameter model was used to calculate the genetic distances between pairs of sequences [41]. The results were used to construct a Neigbour-joining tree using MEGA7. Bootstrapping of 1000 replicates was used to statistically evaluate the branching order of the phylogenetic tree. Bootstrap support of 70% was considered significant and provided evidence for phylogenetic grouping [42]. The phylogenetic tree was rooted using a mongoose rabies biotype.

**Table 2:** The Oligonucleotide primer sequences used in the study showing the annealing positions and their nucleotide sequences [39].

Oligonucleotide	Nucleotide sequence 5’-3’	Position on lyssavirus genome*
550B	GTRCTCCARTTAGCRCACAT	647–666
304	TTGACAAAGATCTTGCTCAT	1514–1533
001lys	ACGCTTAACGAMAAA	1–15

*Nucleotide positions are numbered according to the Pasteur Virus genome sequence.

#### Mouse inoculation test (MIT)

A 10% brain suspension of the six incongruent brain samples was prepared in DMEM/F12 cell culture media, supplemented with 5% gamma irradiated fetal bovine serum (Thermo Fisher Scientific, South Africa), antibiotics (penicillin and streptomycin) and antimycotic (amphotericin B, Sigma-Aldrich, South Africa) before 30 μl of each sample was inoculated into a Balb/C mouse family (each consisting of 8–10 and 2–3 day old suckling mice) (Onderstepoort Biological Products, South Africa) and observed over a 28-day period. The mice were monitored thrice daily for any signs of impaired or sluggish movement and the observations were recorded. After 28 days of observation, all surviving mice were humanely sacrificed by inhalation of isoflurane and lyssavirus infection confirmed or ruled out by testing brains of mice that succumbed during the 28-day period with the DFA assay.

#### Statistical analysis of results

The statistical analysis of the diagnostic efficacy was performed by assuming an exact binomial distribution (MedCalc 12.2.1.0, Ostend, Belgium). The number of true-positive and true-negative samples was determined by the DFA test to establish the sensitivity, specificity, as well as the positive and negative predictive values of the dRIT and qRT-PCR.

## Results

### Cost implications associated with DFA and dRIT implementation

#### Capital investment requirements

Based on the capital investment for each assay, we estimated the costs associated with procuring the DFA equipment to be approximately USD 11,319, while the dRIT equipment amounted to approximately USD 2,069 (Table 3) (See also S1 File. “Supporting document 1” for additional details). The total capital investment for the dRIT assay, calculated over multiple years, remained below that of the DFA assay, irrespective of the period of use (Table 3)(See also S1 File. “Supporting document 1” for additional details).

**Table 3:** Estimated “total cost per sample” for both the direct fluorescent antibody (DFA) and direct, rapid immunohistochemical test (dRIT) assays.

50 samples per annum						
	1 year	1 year	5 years	5 years	10 years	10 years
	DFA	dRIT	DFA	dRIT	DFA	dRIT
Total capital investment	$11 319	$2 069	$2 264	$414	$1 132	$207
Total operational costs	$7 948	$7 951	$10 827	$9 820	$15 023	$12 539
**Total cost per year**	**$19 267**	**$10 020**	**$13 091**	**$10 234**	**$16 155**	**$12 746**
**Total cost per sample**	**$385**	**$200**	**$262**	**$205**	**$323**	**$255**
**500 samples per annum**						
	**DFA**	**dRIT**	**DFA**	**dRIT**	**DFA**	**dRIT**
Total capital investment	$11 319	$2 069	$2 264	$414	$1 132	$207
Total operational costs	$8 928	$8 741	$11 991	$10 758	$16 406	$13 653
**Total cost per year**	**$20 247**	**$10 810**	**$14 255**	**$11 172**	**$17 538**	**$13 860**
**Total cost per sample**	**$40**	**$22**	**$29**	**$22**	**$35**	**$28**
**1000 samples per annum**						
	**DFA**	**dRIT**	**DFA**	**dRIT**	**DFA**	**dRIT**
Total capital investment	$11 319	$2 069	$2 264	$414	$1 132	$207
Total operational costs	$12 488	$12 406	$16 219	$15 110	$21 427	$18 822
**Total cost per year**	**$23 806**	**$14 474**	**$18 483**	**$15 524**	**$22 559**	**$19 029**
**Total cost per sample**	**$23,81**	**$14,47**	**$18,48**	**$15,52**	**$22,56**	**$19,03**

#### Operational costs

The operational costs for each assay were determined for one-, five- and ten-year periods with the findings indicating that the operational costs associated with the dRIT were lower than those for the DFA assay (Table 3) (See S1 File. “Supporting document 1” for additional details).

#### Total cost of diagnosis

Through the work described here, we established the total cost of diagnosis for both the DFA and dRIT assays (Table 3), with the results indicating that the dRIT was cheaper in terms of both “total cost per year” and “total cost per sample diagnosed” under all of the circumstances investigated by the simulation framework (Table 3) (See also S1 File. “Supporting document 1” for additional details).

### Diagnostic efficacy of the DFA and dRIT assays based on panel of samples collected from Nigeria

#### Direct fluorescent antibody test

Of the 278 samples tested with the DFA assay at the NVRI, 23 brain tissue samples (8.3%) collected from dog markets were found to be rabies-positive (Tables 4 and 5). Of these, 23 positive samples, 13/18 (72%) were from suspect animals, while 10/260 (3.8%) were from supposedly healthy animals. Concordant results were obtained when the 28 samples (23 rabies-positive and 5 rabies-negative from the remaining suspect animal samples) were re-tested at the ARC-OVI using the DFA test (Table 5).

**Table 4:** Diagnostic overview of the brain tissue samples tested with the direct fluorescent antibody test in Nigeria, disaggregated by State and sample cohort.

Location (State)	Number of dogs collected from each dog market	Numberof DFA- positive dog brain from each market	Number of rabies-suspect dogs collected from eachdog market	Number of DFA- positive rabies- suspect dogs from each market	Total number of brain tissue samples tested	Number of DFA-positive brain tissue samples	% positive
Anambra	59	1	3	2	62	3	4.8
Ebonyi	62	1	3	1	65	2	3.1
Enugu	139	8	12	10	151	18	11.9
**Total**	**260**	**10**	**18**	**13**	**278**	**23**	**8.3**
**DFA: direct fluorescent antibody test**							

**Table 5:** Neuronal tissue sample cohort from Nigeria depicting the initial diagnostic results from the NVRI in Nigeria and their diagnostic testing at the laboratories in South Africa.

Number	Sample number	DFA (Nigeria)	DFA (South Africa)	DRIT (South Africa)	qRT-PCR (South Africa)
1	49	+	+	+	+
2	50	+	+	+	+
3	65	+	+	+	+
4	95	+	+	+	+
5	140	+	+	+	+
6	142	+	+	+	+
7	176	+	+	+	+
8	185	+	+	+	+
9	249	+	+	+	+
10	251	+	+	+	+
11	261	+	+	+	+
12	262	+	+	+	+
13	263	+	+	+	+
14	264	+	+	+	+
15*	265	-	-	+	+
16	266	+	+	+	+
17	267	+	+	+	+
18	268	+	+	+	+
19	269	+	+	+	+
20	270	+	+	+	+
21*	271	-	-	+	+
22	272	+	+	+	+
23*	273	-	-	+	+
24	274	+	+	+	+
25^#^	275	+	+	+	-
26	276	+	+	+	+
27*	277	-	-	+	+
28*	278	-	-	+	+


Note: “-” represents a rabies-negative diagnosis; “+” represents a rabies-positive diagnosis.

“*” indicates that hemi-nested PCR amplicons were also obtained

“#” indicates that the hemi-nested PCR was applied but that no amplicons were obtained.

DFA: direct fluorescent antibody test

dRIT: direct, immunohistochemical test

qRT-PCR: quantitative real-time polymerase chain reaction

#### Direct, rapid immunohistochemical test (dRIT)

The dRIT assay found that all of the brain tissue samples (*n = 23* DFA-positive and *n = 5* DFA-negative) were lyssavirus-positive (Table 5). These results, however, were discordant to those observed for the DFA assay implemented in both Nigeria and South Africa.

#### Quantitative real-time reverse transcription polymerase chain reaction

Using the qRT-PCR, viral RNA was detected and amplified in all the brain tissue samples–with the exception of one (275NG)–indicating the presence of lyssavirus genomic viral RNA in 27/28 brain samples (Table 5).

#### Sensitivity and specificity of the DFA, hn-PCR and dRIT

Based on the fact that the DFA is considered the gold standard test, the DFA assay was used as the reference test to which the two other OIE-recommended assays (DRIT and qRT-PCR) were statistically compared (Table 6). In instances where any incongruent results were observed the hn-PCR assay was used for additional diagnostic confirmation.

**Table 6:** Diagnostic sensitivity and specificity of the three OIE recommended diagnostic assays applied to a cohort of samples collected from dog meat markets.

DFA (Nigeria and South Africa)*							
True positive	False positive	True negative	False negative	Diagnostic sensitivity	Diagnostic specificity	Positive predictive value	Negative predictive value
23	0	5	0	100%	100%	100%	100%
dRIT (South Africa)							
23	5	0	0	100%	0.0%	82.1%	0.0%
qRT-PCR (South Africa)							
23	4	0	1	100%	0.0%	85.2%	0.0%

* The DFA was used as the gold standard and reference test in the analyses

#### Hemi-nested (hn) PCR

**Fig 3:**
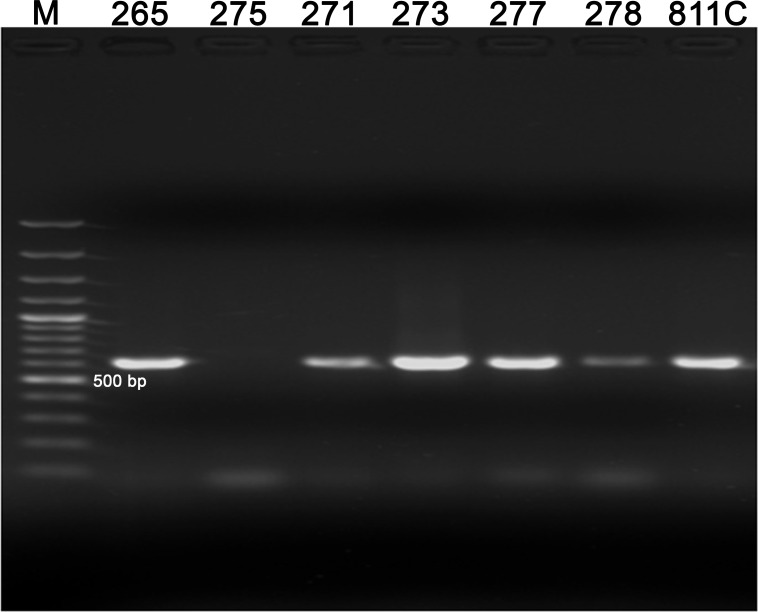
Hemi-nested PCR showing amplification products of approximately 606 bp following agarose gel electrophoresis. *M– 100 bp Molecular weight marker (Promega); C- Negative control; 811/97—Positive control.

The hn-PCR amplified nucleic acids with the expected band size (approximately 660 bp) for all five discrepant DFA-negative samples (Table 5). The remaining discrepant sample (275NG; negative in the qRT-PCR) did not yield an amplicon with the PCR (Fig 3), but interestingly was positive with both the DFA and the dRIT assays (Table 4).

#### Phylogenetic analysis of the five discrepant brain samples

**Fig 4:**
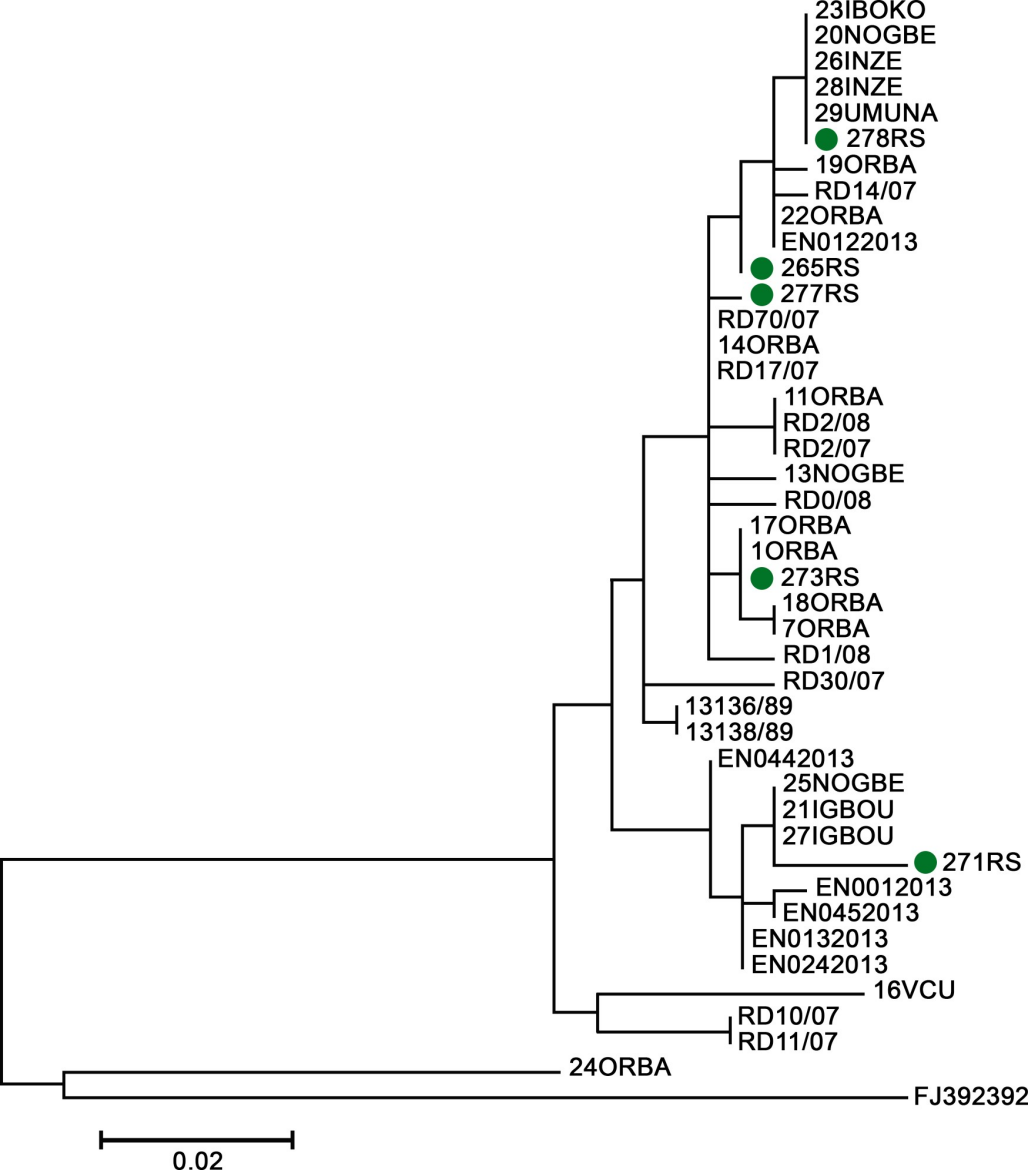
Molecular phylogenetic analysis by neighbour joining method showing the five samples (green dots) with discordant DFA, dRIT and hn-qPCR results forming part of the canine variant of RABV that clustered with other Nigerian RABVs.

The nucleic acid of the five samples that amplified with the hn-PCR reaction were subsequently subjected to Sanger sequencing in order to rule out the possibility of laboratory contamination (Fig 4)

#### Mouse inoculation test (MIT)

In the MIT assay, mouse deaths were recorded in families inoculated with 271NG (*n = 1*), 277NG (*n = 5*) and 278NG (*n = 3*), but none of these deaths were attributable to lyssavirus infection through DFA testing.

## Discussion

South-East Nigeria has at least one major dog market and other smaller dog markets in each of the States. These typically unregulated markets have ongoing prohibited activities including the illegal importation and sale of free-roaming dogs from neighboring countries [21, 43, 44], increasing the potential risk for cross-border transmission of rabies and other transboundary diseases such as foot and mouth disease (FMD) and peste des petit ruminants (PPR) [45, 46]. Such a disease transmission pattern makes dog markets a locus for rabies outbreaks given that there are no current government policies regulating dog meat trade. Indeed, the preliminary results of our study implied that some of the dogs sold in the dog markets in South-East Nigeria were lyssavirus-infected, and that the prevalence of rabies in the Southern States appeared to be relatively low when compared to those in Northern States [14,15,16]. This finding is consistent with those of other studies investigating rabies prevalence in Nigerian dog meat markets (1.58% - 31.0%), suggesting that rabies is a potential health risk to the people processing dog meat within such an environment [14, 16, 17, 18, 19, 20, 21].

Since there is no laboratory capable of undertaking rabies diagnosis in South-East Nigeria, all rabies suspect cases are currently sent to the NVRI in northern Nigeria for diagnostic testing–hindering the regularity of routine rabies surveillance and preventing the true burden from being ascertained. In order to improve rabies surveillance within these dog meat markets and in Nigeria in general, active disease surveillance relying on decentralized or field-based rabies testing is required [47].

To this end, we undertook a cost analysis to determine the total cost of implementation of routine rabies diagnosis using either the dRIT or DFA diagnostic assays where both capital investments and running costs were considered. The findings of our investigation indicated that the total cost associated with the dRIT would be between ±47% and ±16% cheaper–depending on the number of samples diagnosed per annum (See Table 3 and S1 File. “Supporting document 1” for additional details). As such, it would make practical and financial sense to implement decentralized rabies diagnosis using the dRIT assay, focusing not only on the dog meat markets of Nigeria, but also for field-based surveillance in other parts of the continent.

Furthermore, we compared the diagnostic performance of three diagnostic assays that are currently recommended by the OIE for rabies diagnosis (DFA, dRIT and qRT-PCR). In this assessment, 28 samples collected from Nigerian dog meat markets were sent to South Africa for confirmatory rabies diagnosis. In South Africa, the DFA assay produced concordant diagnostic outcomes in the 28 brain tissue samples tested in Nigeria, while the statistical analysis suggested that the dRIT and qRT-PCR produced various false-positive results when applied to the same panel of samples (Table 6). It could, however, be argued that the DFA reference test produced false-negative results rather than the contrary. Indeed, the DFA in both Nigeria and South Africa diagnosed the same five samples as rabies-negative whilst these were shown to be lyssavirus positive when tested with the dRIT, qRT-PCR and hn-PCR–suggesting that the samples contained both viral antigen and nucleic acid. These discrepancies underscore the need for diagnostic laboratories to include confirmatory methods in their diagnostic regime in order to provide accurate data and continuously validate their diagnostic methods.

We speculate that the false-negative outcome provided by the DFA in our evaluation could be explained by its limitation to detect very low numbers of viral antigen in tissues [48]. This theory was supported by the qRT-PCR results of these samples that demonstrated low RNA copy numbers in the brain specimens that gave incongruent results (see S2 File. “supporting document 2”). The five samples that gave discordant results were also further subjected to Sanger sequencing which demonstrated that the five viruses all formed part of the canine variant of RABV that clustered with other Nigerian RABVs (Fig 4)–eliminating the suspicion of potential laboratory contamination during molecular testing.

While the results from the qRT-PCR were almost identical to those produced by the dRIT, one sample [275NG] that was both DFA and dRIT positive did not have amplified nucleic acid (Table 5). The sample in question, was however, still deemed rabies-positive as both the DFA and dRIT demonstrated the presence of lyssavirus antigen. As such, we speculate that the viral RNA of this sample could have been degraded either during or prior the total RNA extraction steps. Unfortunately, the MIT could not provide additional clarity as the MIT results were negative, supporting the lack of viable or replicating virus in the samples in question.

An alternative explanation could be the conjugates that were used for DFA diagnosis in South Africa and Nigeria. Published findings from inter-laboratory trials using the DFA have found that the conjugates used by different laboratories influenced the diagnostic sensitivity of the DFA test applied to the same panel of samples [49, 50, 51].

Regardless of the specific reasons for the reduced diagnostic sensitivity, our results suggest that the DFA assay applied in Nigeria could have misdiagnosed many more rabies-positive samples during the initial round of screening of samples collected from the dog meat markets. These findings, although not unique to Nigeria, illustrate how reduced diagnostic proficiency results in an underestimation of the true burden of rabies on the African and Asian continents. This is a problem that can be addressed by utilizing setting-specific diagnostic assays (especially in resource-limited settings) and implementing a confirmatory method for lyssavirus diagnosis. Considering that rabies infection involving human exposures to rabies suspect animals can only be ruled out after confirmatory testing, it would be prudent to establish centralized confirmatory testing ideally using an assay relying on nucleic acid amplification (e.g. qRT-PCR) in Nigeria. This would allow the reference and decentralized laboratories to continually validate the DFA or dRIT results in order to assess their reliability in various settings. Furthermore, participation in regular international proficiency testing through inter-laboratory trials would be pertinent; not only for Nigeria, but for all rabies-endemic countries, ensuring that staff are adequately trained and that the diagnostic tests are suitably and accurately undertaken.

We acknowledge that there were several limitations associated with this study, including: 1) the limited information known about dog meat markets due to poor legislation; 2) the limited cohort of samples and the challenge of adequate, unbiased representation; 3) the remaining 250 samples that tested negative in Nigeria were not re-tested for confirmation in another laboratory, potentially resulting in further missed rabies cases. The third limitation mentioned has the resultant effect of introducing bias into the sensitivity and specificity values for this study. We attempted to garner as much information as possible for every sample and attempted to reduce any bias through the use of multiple diagnostic assays on every sample.

### Conclusions

Accurate and decentralized rabies diagnosis in the six geopolitical zones of Nigeria (with the NVRI acting as the national reference laboratory) will aid in the prompt diagnosis, monitoring and reporting of rabies cases throughout the country–ensuring an improved burden-estimate throughout the country. Therefore, it will be particularly beneficial if rabies diagnosis is decentralized to include the dRIT testing, enabling every state to perform primary diagnosis before sending the samples to the reference laboratories for confirmatory testing should the need arise. These findings demonstrated that the dRIT is capable of detecting low rabies-positive samples, supporting previous reports that the dRIT assay is not only extremely effective, but also reliable and well-suited to resource-limited settings such as Nigeria where sample conditions will not always be optimal [26, 27, 31, 32,33,52]. Coupled with this, the cost of the routine use of the dRIT makes this assay ideal for decentralized rabies diagnosis in resource-limited settings such as Nigeria.

By implementing a feasible, accurate and cost-effective diagnostic assay in decentralized manner, rabies surveillance in Nigeria can be drastically improved, enabling the government to ascertain the true burden of rabies in Nigeria, break the cycle of neglect, and adequately prevent more dog-mediated human rabies deaths, a move likely to achieve zero human deaths by 2030.

## Supporting information

S1 File Estimating the potential cost of implementing rabies diagnostic assays in developing countries. (PDF)

S2 File Neuronal tissue sample cohort from Nigeria depicting the estimated viral RNA copy numbers as determined using a quantitative real-time polymerase chain reaction assay. (DOCX)
