# Supplementary material for: Economic and feasibility comparison of the dRIT and DFA for decentralized rabies diagnosis in resource-limited settings: The use of Nigerian dog meat markets as a case study
Source: PLoS Negl Trop Dis. 2020 Feb 28;14(2):e0008088. doi: 10.1371/journal.pntd.0008088 (PMC7065817; doi:10.1371/journal.pntd.0008088)
Supplement: S1 File — (PDF) [file pntd.0008088.s001.pdf]

Estimating the potential cost of implementing rabies diagnostic assays in developing countries

Andre Coetzer, Terence P. Scott, Claude T. Sabeta and Louis H. Nel

Submitted as Supporting Information

1. Introduction

Diagnostic capacity is critical to disease surveillance and generating accurate data pertaining to disease outbreaks and epidemics. As such, increasing diagnostic capacity is vital if the cycle of neglect that hinders governmental buy-in and support is to be broken [1]. For rabies specifically, the World Organisation for Animal Health (OIE) has recently updated its guidelines in which it now recognises and recommends several diagnostic assays for the purpose of rabies diagnosis [2]. The option of implementing a variety of diagnostic assays has left governmental stakeholders with a number of assays to choose from, with the applicability of a diagnostic assay primarily being driven by the statistical relevance (diagnostic sensitivity and diagnostic specificity) of the given test [3]. Additional factors that influence the general implementation of the respective assay are, however, often not considered fully in the published literature. Indeed, apart from the statistical significance of a given test, the cost associated with the establishment of a diagnostic facility and undertaking routine diagnosis over a set time period cannot be overlooked when considering the applicability of a diagnostic assay – especially in resource-limited countries in Asia and Africa. Without such information, governmental stakeholders may make uninformed decisions as to the implementation of a diagnostic assay in their jurisdiction that may not be best suited to their financial and developmental needs, especially considering the highly comparable statistical significance of the tests that are recognised by the OIE.

To this end, we developed a simulation framework that considered the costs associated with the routine implementation of the direct fluorescent antibody (DFA) and the direct, rapid immunohistochemical test (dRIT) assays for rabies diagnosis in an simulated diagnostic facility assuming a basic pre-existing infrastructure. Even though it is an OIE-recommended assay, the routine implementation of the quantitative real-time polymerase chain reaction (qRT-PCR) was not included in our evaluation as it would require considerable capital investment in terms of equipment, training and the facility modification required to establish clean rooms – making it impractical for most resource-limited countries.

We considered the capital investment and the operational costs associated with routinely performing either the DFA or the dRIT diagnostic assay. The resulting cost estimations were

used to predict the financial outcomes in low-, medium- and high-throughput facilities over the course of differing periods of time to determine which of the two diagnostic assays would be more viable financially and thus preferable in terms of implementation in resource-limited settings.

2. Materials and methods

2.1. Description of the simulation framework

To estimate the costs associated with implementing either the DFA or dRIT assay, a simulation framework was developed based on a simulated resource-limited country. This cost estimation was based on the assumption that the facilities containing the basic infrastructure (e.g. laboratory equipment, electricity, water, etc.) would be available in the modelled country. Furthermore, we relied on two data sets: i) laboratory throughput (based on three throughput scenarios, i.e. 50, 500 and 1000 samples per annum) and; ii) cost data (based on both capital investment and operational costs calculated over a one-, five- and ten-year period).

2.1.1. Laboratory throughput

When considering laboratory throughput, the financial implications of diagnosing a low (*n = 50* samples per annum), medium (*n = 500* samples per annum) or high (*n = 1000* samples per annum) number of samples over a period of multiple years was considered. In addition to the three theoretical throughput rates per annum, the average number of samples per day was calculated based on the average number of working days per annum in the modelled country.

2.1.2. Cost data

In our investigation we considered the capital investment and operational costs to obtain a clearer representation of the various financial components associated with each test as well as the impact on the price per diagnostic reaction.

2.2. Capital investment

The capital investment consisted of all the costs that were directly associated with procuring the equipment required for each diagnostic assay. These costs consisted of equipment common to both diagnostic assays (e.g. fridge/freezer combination, forceps, scissors, glass submersion chambers, humidity boxes and a pipettor) and equipment that was unique to each assay such as the fluorescent microscope and incubator for the DFA test and a compound light microscope for the dRIT assay. Here we utilised only the lowest possible price for the

equipment (permitting that it would be of adequate quality for use during rabies diagnosis) that was unique to each of the two diagnostic tests to account for facilities with a low start-up capital. Furthermore, we implemented a multi-year analysis to account for equipment investment as it was unlikely that a government would invest the money as a single year investment. In the multi-year analysis, the equipment costs were calculated over a one-year, five-year and ten-year period to enable an accurate calculation of the “cost per diagnostic reaction” that considered all relevant contributing factors.

2.3. Operational costs

For our investigation, the operational cost was further split into fixed and variable costs. The costs of facilities, cleaning staff and communications were not factored into our analysis as the cost estimates were based on the use of pre-existing diagnostic facilities. In addition, smaller sundry operational costs such as insurance were considered, but in the final analysis the impact of such indirect costs were insignificant and thus excluded.

2.3.1. Fixed costs

The fixed costs attributed to each of the two diagnostic assays in our investigation were the labour costs associated with a laboratory diagnostician, cost of the annual microscope service and the vaccination of the diagnostic technician. In an effort to make the fixed costs comparable to the capital costs that were depreciated over multiple years, the fixed costs were also calculated across a similar timeframe while taking the following factors into consideration: i) an average annual inflation rate for the labour cost was assumed to be 3.5% [4], ii) diagnostic technicians would require two doses of rabies vaccine in the first year and an annual booster consisting of one dose of vaccine per year thereafter and; iii) the annual servicing (adjusted by taking a 3.5% annual inflation into consideration [4]) of all microscopes is required.

2.3.2. Variable costs

The variable costs included those associated with the diagnostic reagents and consumables that would be required to implement either of the two assays under investigation. Furthermore, the variable costs considered the direct influence of the number of samples subjected to a single diagnostic run and how the reagents and consumables were applied, e.g. touch impressions of samples on a slide were either covered with a specific reagent (lower reagent volume) or submerged in a glass submersion chamber (higher reagent volume, but permits multiple uses for high-throughput). In addition, we calculated the total variable cost per annum

by multiplying the calculated reagent cost per run with the number of samples diagnosed per year. Lastly, we implemented a multi-year analysis for the cost of the reagents and consumables, taking an average annual inflation rate of 3.5% into consideration [4].

2.4. Total cost of diagnosis

To determine the total cost per diagnostic assay for both the DFA and dRIT assays, we divided the final cost (consisting of both capital investment and operational costs) by the average number of samples tested per year. In addition, the total cost of rabies diagnosis was considered for a one-, five- and ten-year period to account for a multi-year investment in the decentralised diagnostic facility.

3. <u>Results</u>

3.1. Determination of the theoretical laboratory throughput

To determine the theoretical laboratory throughput, we assumed that each year consists of 250 working days. Days excluded from the 365 days per annum included weekends (n = 104 weekend days) and the average number of public holidays in developing countries in Africa (*n = 11*) [6] (Table S1).

**Table S1. Average diagnostic laboratory throughput**			
**Throughput classification**	**Average number of samples per annum**	**Average number of samples per five day work week**	**Average number of samples per day**
**Low**	50	1	0,2 *
**Medium**	500	10	2
**High**	1000	20	5
* One sample on one day of the five-day work week			

3.2. The capital investment required to implement the two diagnostic assays

Based on the capital investment for each assay, we estimated the costs associated with procuring the DFA equipment to be approximately USD 11,319, while the dRIT equipment amounted to approximately USD 2,069 (Table S2). The total capital investment for the dRIT assay, calculated over multiple years, remained below that of the DFA assay, regardless of the period of use (Table S2).

Table S2. Capital investment required to implement each of the diagnostic assays		
**Common equipment**	***DFA (USD)***	***dRIT (USD)***
Refrigerator/Freezer unit	$179	$179
Forceps and Scissors	$25	$25
Glass slide submersion chamber	$3	$3
Humidity chamber	$37	$37
Clock timer	$25	$25
Pipettor (200 ul)	$300	$300
**Sub total:**	**$569**	**$569**
**Unique equipment**	***DFA (USD)***	***dRIT (USD)***
37 °C Incubator	$750	---
Appropriate microscope	$10,000	$1,500
**Sub-total:**	**$10,750**	**$1,500**
**Total equipment cost (Year 1):**	**$11 319**	**$2 069**
**Total equipment cost (Year 5):**	**$2 264**	**$414**
**Total equipment cost (Year 10):**	**$1 132**	**$207**

3.3. Determination of the operational costs associated with the two diagnostic assays

3.3.1. Fixed costs associated with the two diagnostic assays

The fixed costs for each assay were determined for a one-, five- and ten-year period with the findings indicating that the fixed costs associated with the dRIT were lower than those for the DFA assay (Table S3). While the cost of employing a diagnostician – and ensuring that they are adequately vaccinated – was the same for both assays, the estimated cost associated with the annual servicing of the microscope was significantly cheaper for the compound light microscope used in the dRIT (Table S3).

Table S3. Multi-year analysis of total fixed costs associated with the two assays

DFA	Year 1	Year 5	Year 10
Annual cost of laboratory diagnostician [#]	$7 670	$8 800	$10 450
Annual vaccination of diagnostic technician	$80	$254	$509
Servicing of fluorescent microscope [+]	$0	$1 538	$3 785
**Total fixed cost per annum**	**$7 750**	**$10 592**	**$14 744**
**dRIT**	**Year 1**	**Year 5**	**Year 10**
Annual cost of laboratory diagnostician [#]	$7 670	$8 800	$10 450
Annual vaccination of diagnostic technician	$80	$254	$509
Servicing of light microscope [+]	$0	$527	$1 296
**Total fixed cost per annum**	**$7 750**	**$9 581**	**$12 255**

[#] While the labour cost for a laboratory technician would vary between resource limited countries in Africa, an average annual salary for a diagnostic technician in South Africa was used as an upper estimate of what the cost would be per year [5].
+ We assumed that a microscope would not be serviced in its first year of use.

3.3.2. Variable costs associated with the two diagnostic assays

The reagent and consumable costs required to perform either of the diagnostic assays were determined based on lowest available cost estimates and did not include transport costs from the supplier to the end-user (Table S4 and Appendix 1). Despite requiring more reagents per run to undertake the dRIT, the reagent costs associated with the DFA and dRIT assays were comparable under most circumstances. Indeed, the dRIT reagents were marginally cheaper for all but one of the modelled laboratory throughputs (1 slide per run) (Table S4). This observation was found to be true even when the variable costs were considered over multiple years (Table S5).

Table S4. Reagent and consumables cost associated with the two diagnostic assays in laboratories with a low-, medium- and high-throughput			
**DFA**			
**Reagent**	**1 slide per run**	**2 slides per run**	**5 slides per run**
1) FITC labelled antibody	$0,0014	$0,0014	$0,0014
2) 100% acetone	$3,24	$1,62	$0,65
3) 1xPBS	$0,38	$0,19	$0,08
4) Microscope slide	$0,06	$0,06	$0,06
5) Cover slip	$0,04	$0,04	$0,04
6) Consumables	$0,23	$0,44	$1,07
**Total reagent cost**	**$3,96**	**$2,36**	**$1,90**
**Total reagent cost per annum**			
50 samples per annum	$198		
500 samples per annum		$1 178	
2500 samples per annum			$4 738
**dRIT**			
**Reagent**	**1 slide per run**	**2 slides per run**	**5 slides per run**
1) Biotinylated Ab	$0,0036	$0,0036	$0,0036
2) Formalin	$0,85	$0,43	$0,17
3) 3% H2O2	$0,06	$0,03	$0,01
4) Streptavidin	$0,13	$0,13	$0,13
5) AEC chromogen	$0,06	$0,06	$0,06
6) Gills #2 formula	$1,30	$0,13	$0,07
7) 1xPBS	$1,17	$0,60	$0,23
8) Tween80	$0,12	$0,06	$0,02
9) Microscope slide	$0,06	$0,06	$0,06
10) Cover slip	$0,04	$0,04	$0,04
11) Consumables	$0,23	$0,44	$1,07
**Total reagent cost**	**$4,02**	**$1,98**	**$1,86**
**Total reagent cost per annum**			
50 samples per annum	$201		
500 samples per annum		$991	
2500 samples per annum			$4 656

Table S5. Multi-year analysis of reagent and consumables cost associated with the two assays									
Assay	***Year 1***			***Year 5***			***Year 10***		
	**50 samples per annum**	**500 samples per annum**	**1000 samples per annum**	**50 samples per annum**	**500 samples per annum**	**1000 samples per annum**	**50 samples per annum**	**500 samples per annum**	**1000 samples per annum**
DFA	$198	$1 178	$4 738	$235	$1 399	$5 627	$279	$1 662	$6 683
dRIT	$201	$991	$4 656	$239	$1 177	$5 529	$284	$1 398	$6 567

3.4. Total cost of diagnosis

Through the work described here, we established the total cost of diagnosis over time for both the DFA and dRIT assays (Table S6), with the results indicating that the dRIT was the cheaper option (based on both "total cost per year" and "total cost per sample diagnosed") under all of the circumstances investigated by the simulation framework (Figure S1 – S3).

Table S6. Total cost of diagnosis						
**50 samples per annum**	**DFA**	**dRIT**	**DFA**	**dRIT**	**DFA**	**dRIT**
	**1 year**	**1 year**	**5 years**	**5 years**	**10 years**	**10 years**
Total capital investment	$11 319	$2 069	$2 264	$414	$1 132	$207
Total operational costs						
- Fixed costs	$7 750	$7 750	$10 592	$9 581	$14 744	$12 255
- Variable costs	$198	$201	$235	$239	$279	$284
**Total cost per year**	**$19 267**	**$10 020**	**$13 091**	**$10 234**	**$16 155**	**$12 746**
**Total cost per sample**	**$385**	**$200**	**$262**	**$205**	**$323**	**$255**
**Percentage difference**	**dRIT 48% cheaper**		**dRIT 22% cheaper**		**dRIT 21% cheaper**	
[redacted]						
**500 samples per annum**	**DFA**	**dRIT**	**DFA**	**dRIT**	**DFA**	**dRIT**
	**1 year**	**1 year**	**5 years**	**5 years**	**10 years**	**10 years**
Total capital investment	$11 319	$2 069	$2 264	$414	$1 132	$207
Total operational costs						
- Fixed costs	$7 750	$7 750	$10 592	$9 581	$14 744	$12 255
- Variable costs	$1 178	$991	$1 399	$1 177	$1 662	$1 398
**Total cost per year**	**$20 247**	**$10 810**	**$14 255**	**$11 172**	**$17 538**	**$13 860**
**Total cost per sample**	**$40**	**$22**	**$29**	**$22**	**$35**	**$28**
**Percentage difference**	**dRIT 47% cheaper**		**dRIT 22% cheaper**		**dRIT 21% cheaper**	
[redacted]						

**1000 samples per annum**	**DFA**	**dRIT**	**DFA**	**dRIT**	**DFA**	**dRIT**
	**1 year**	**1 year**	**5 years**	**5 years**	**10 years**	**10 years**
Total capital investment	$11 319	$2 069	$2 264	$414	$1 132	$207
Total operational costs<br>- Fixed costs<br>- Variable costs	$7 750<br>$4 738	$7 750<br>$4 656	$10 592<br>$5 627	$9 581<br>$5 529	$14 744<br>$6 683	$12 255<br>$6 567
**Total cost per year**	**$23 806**	**$14 474**	**$18 483**	**$15 524**	**$22 559**	**$19 029**
**Total cost per sample**	**$23,81**	**$14,47**	**$18,48**	**$15,52**	**$22,56**	**$19,03**
**Percentage difference**	**dRIT 39% cheaper**		**dRIT 16% cheaper**		**dRIT 16% cheaper**	

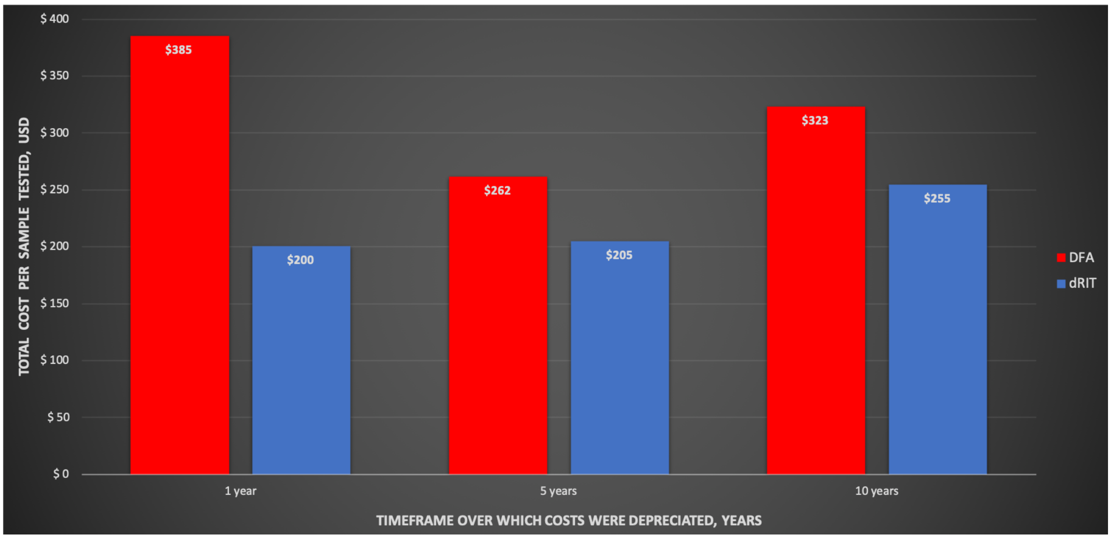

Figure S1. Total cost per sample diagnosed with either the DFA or dRIT assay (50 samples per annum)

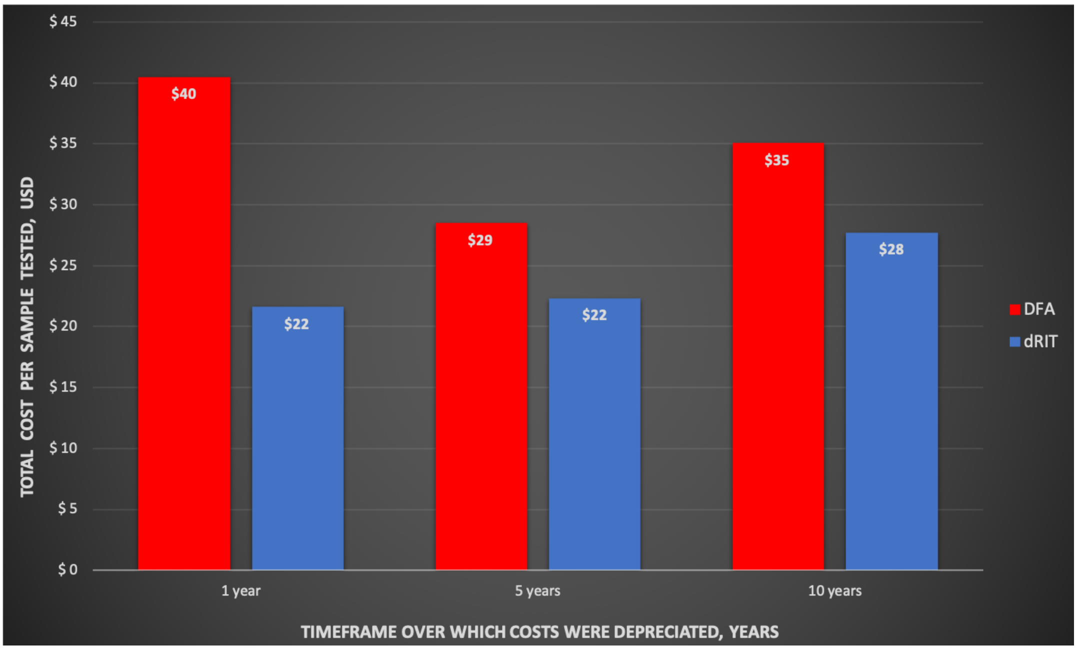

Figure S2. Total cost per sample diagnosed with either the DFA or dRIT assay (500 samples per annum)

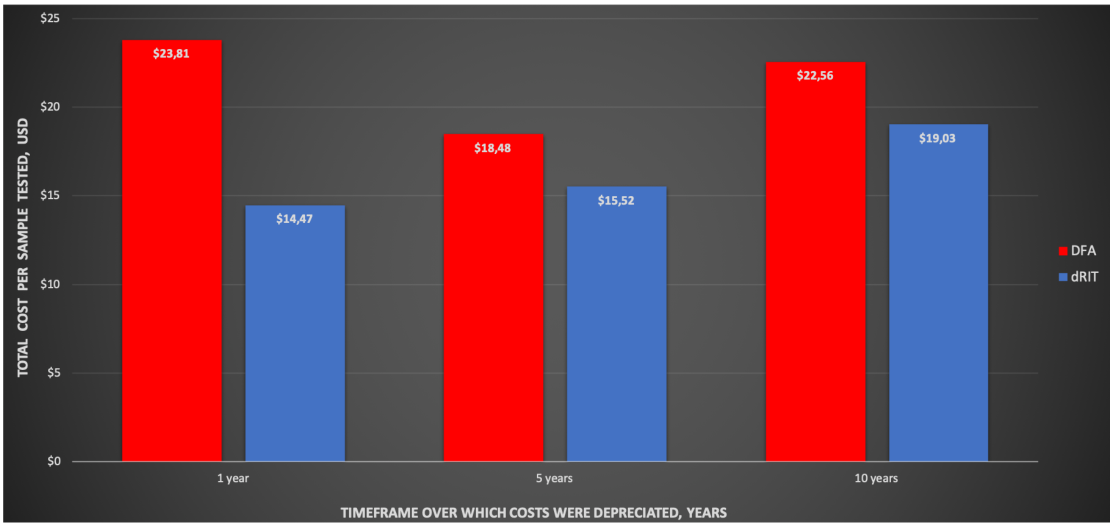

Figure S3. Total cost per sample diagnosed with either the DFA or dRIT assay (1000 samples per annum)

4. Conclusion

To date, comparative studies between the DFA and dRIT have suggested that the dRIT would be cheaper to implement or decentralise based on the fact that the dRIT only requires a compound light microscope [8,9]. As shown in this evaluation, the compound light microscope is indeed a less expensive item of equipment compared to its fluorescent counterpart, but only accounts for one of the many associated costs for rabies diagnosis. To consider all the variables, we analysed all relevant costs when implementing either the DFA or dRIT assay in a modelled developing country. To account for the varying number of samples that could be received every year, three theoretical throughput rates (50, 500 and 1000 samples per annum) were used to predict the effect that the varying number of samples would have on the price per diagnosis.

The initial capital investment required to set up a diagnostic facility for each of the methodologies differed significantly between the two OIE-recommended assays (DFA: ±USD 11,319 vs dRIT: ±USD 2,069) (Table S2). This difference was due to the high cost of the fluorescent microscope for the DFA diagnostic test, while the dRIT test required only a high quality compound light microscope.

The fixed costs considered the costs of employing a diagnostician, their pre-exposure prophylaxis and the annual servicing of equipment (Table S3). These fixed costs were included as they are critical to safe and effective diagnostic practices. Indeed, without ensuring the safety of the employed diagnostician and ensuring the optimal working order of the equipment, the diagnostic proficiency could deteriorate or lapse entirely, resulting in a major impact on the surveillance network and overall elimination strategies [10].

The variable costs considered in our investigation were the costs associated with the reagents and consumables. As the DFA test required significantly fewer reagents (Appendix materials), the total reagent cost per annum for the DFA was expected to be lower. This was only true in low-throughput laboratories with marginal differences, whilst the dRIT was marginally cheaper in higher throughput laboratories (Table S4). The higher costs in low-throughput laboratories for the dRIT could be explained by the fact that this assay relies on more steps where the tissue impression is submerged in a large volume of reagent, with the number of slides submerged in the reagent determining how the cost of the full volume of the reagent is fractioned (See appendix materials). The only variable cost that was not included in the simulation framework was the cost associated with transporting samples to the diagnostic facility. Despite this cost being a major contributing factor to costs associated with rabies diagnosis in any country, it would be the same for either test – resulting in a value that would not contribute to the difference observed in the "cost per diagnostic test".

The primary outcome of our investigation was an ability to factor in all of the pertinent costs and obtain an estimated "cost per diagnostic test" for both the DFA and dRIT assays. Throughout our evaluation, we estimated that the dRIT would be between 47% and 16% cheaper – depending on the number of samples diagnosed per annum (Table S6). In addition, we highlighted the high "cost per diagnostic test" in the low-throughput scenarios (approximately 50 samples per annum) (Figure S1). Those circumstances highlight the potential reasons for limited routine rabies diagnosis in resource-limited developing countries as the number of samples tested in a facility has a significant effect on the cost per test as a result of the relatively high operational costs involved in implementing diagnostic assays.

The findings of our investigation support the view that the dRIT assay (compared to the DFA assay) is financially more feasible in terms of establishment and routine implementation in resource-limited countries. These findings, coupled with the fact that the diagnostic efficacy of the dRIT is comparable to the DFA, suggests that the dRIT has a significant advantage over the DFA for routine implementation within rabies-endemic countries – in so doing contributing valuable surveillance data and breaking the cycle of neglect.

5. References

1. Nel LH. Discrepancies in data reporting for rabies, Africa. Emerg Infect Dis. 2013;19: 529–533.
2. OIE. Rabies (Infection with Rabies Virus and Other Lyssaviruses). In: Manual of Diagnostic Tests and Vaccines for Terrestrial Animals [Internet]. 2018 [cited 26 Jun 2018] pp. 1–35. Available: http://www.oie.int/fileadmin/Home/eng/Health_standards/tahm/2.01.17_RABIES.pdf

3. OIE. Principles and methods of validation of diagnostic assays for infectious diseases. 6th ed. Commission OIEBS, editor. Manual of diagnostic tests and vaccines for terrestrial animals (2010). France: Office International des epizooties; 2008.
4. Statista. Global inflation rate from 2012 to 2022 (compared to previous year) [Internet]. 2019 [cited 31 May 2019]. Available: https://www.statista.com/statistics/256598/global-inflation-rate-compared-to-previous-year/
5. Indeed. Laboratory Technician Salaries in South Africa [Internet]. 2019.
6. AppStudio. African National Public Holidays [Internet]. 2019.
7. WHO. Rabies vaccines: WHO position paper – April 2018. Wkly Epidemiol Rec / Elev épidémiologique Hebd. 2018;16: 201–220. doi:10.1016/j.actatropica.2012.04.013.
8. Dürr S, Naïssengar S, Mindekem R, Diguimbye C, Niezgoda M, Kuzmin I, et al. Rabies diagnosis for developing countries. PLoS Negl Trop Dis. 2008;2: e206. doi:10.1371/journal.pntd.0000206
9. Coetzer A, Anahory I, Dias PT, Sabeta CT, Scott TP, Markotter W, et al. Enhanced diagnosis of rabies and molecular evidence for the transboundary spread of the disease in Mozambique. J S Afr Vet Assoc. 2017;88. doi:10.4102/jsava.v88i0.1397
10. Lechenne M, Naissengar K, Lampelletier A, Alfarouk IO, Bourhy H, Zinsstag J, et al. Validation of a Rapid Rabies Diagnostic Tool for Field Surveillance in Developing Countries. PLoS Neglected Trop Dis. 2016; 1–16. doi:10.1371/journal.pntd.0005010

6. Appendix materials

Average price of reagents per diagnostic run of the DFA diagnostic assay

A1) Fluoroag™ FIC conjugation kit @ USD370 + Unlabelled antibody preparation @ USD 55 = USD 425

Each kit contains a single spin column that produced 3 ml of FITC labelled antibody. The spin column can be re-used five times to supply 15 ml FITC labelled antibody per kit. The antibody working dilution (1:1000) produced 15 000 ml of FITC-labelled antibody at the working concentration. Each slide (containing one touch impression) required approximately 0.05 ml of FITC-labelled antibody per slide.

$$\frac{\text{15 000 ml FITC-labelled antibody per kit}}{\text{0,05 ml FITC-labelled antibody oer slide}} = \text{300 000 slides per kit}$$

$$\frac{\text{USD 425 per kit}}{\text{300 000 slides per kit}} = \textbf{\$0,0014 per sample}$$

A2) 2.5 L 100% Acetone @ USD 55

$$\frac{\text{2 500 ml per bottle of 100\% acetone}}{\text{140 ml acetone per diagnostic run}} = \text{17 runs per bottle}$$

$$\frac{\text{USD 55 per 2,5 L of 100\% acetone}}{\text{17 diagnostic runs per bottle}} = \text{\$3,24 per 140 ml of 100\% acetone}$$

1 Sample per run – Low throughput facility	2 Samples per run – Medium throughput facility	5 Samples per run – High throughput facility
USD 3,24 per 140 ml	USD 3,24 per 140 ml	USD 3,24 per 140 ml
1 slide per 140 ml<br>=<br>**$3,24 per sample**	2 slides per 140 ml<br>=<br>**$1,62 per sample**	5 slides per 140 ml<br>=<br>**$0,65 per sample**

A3) 1L 10xPBS @ USD 27

The standard operating procedure for the DFA diagnostic assay required the use of 1xPBS.

$$\frac{\text{10 000 ml 1x PBS per bottle}}{\text{140 ml 1x PBS per diagnostic run}} = \text{71 runs per bottle}$$

$$\frac{\text{USD 27 per 10L of 1xPBS}}{\text{71 runs per bottle}} = \text{\$ 0,38 per 140 ml of 1xPBS}$$

1 Sample per run – Low throughput facility	2 Samples per run – Medium throughput facility	5 Samples per run – High throughput facility
USD 0,38 per 140 ml	USD 0,38 per 140 ml	USD 0,38 per 140 ml
1 slide per 140 ml<br>=<br>**$0,38 per sample**	2 slides per 140 ml<br>=<br>**$0,19 per sample**	5 slides per 140 ml<br>=<br>**$0,08 per sample**

A4) Microscope slides (pack of 50) @ USD 3

$$\frac{\text{USD 3 per pack}}{\text{50 slides per pack}} = \textbf{\$0,06 per sample}$$

A5) Cover slips (pack of 100) @ USD4,20

$$\frac{\text{USD 4,20 per pack}}{\text{100 cover slips per pack}} = \textbf{\$0,04 per sample}$$

Average price of reagents per diagnostic run of the DFA diagnostic assay					
B1)	Nitrile gloves (box of 100) @ USD 6,50 per box				
	Two pairs of gloves (n = 4) required per diagnostic run according to the double glove policy				
	100 gloves per box / USD 6.5 per box		=	$0,0065	per glove
	$0,0065	x	4	=	**$0,0260** per run (regardless of sample number)
B2)	Pipettor tips (1 – 200 ul) @ USD 10 for 96 tips				
	2 tips required per sample (1 to add antibody and 1 to add mounting media to the impression				
	USD 10 per tray / 96 tips per tray		=	**$0,10**	**per tip**

1 Sample per run – Low throughput	2 Samples per run – Medium	5 Samples per run – High
$0,1042 x 2	$0,1042 x 4	$0,1042 x 10
=	=	=
**$0,21 per run**	**$0,42 per run**	**$1,04 per run**

Average price of reagents per diagnostic run of the dRIT diagnostic assay

C1) EZ-Link® Sulfo-NHS-Biotinylation Kit @ USD 380 + Unlabelled anti-ribonucleoprotein polyclonal antibody @ USD 55 = USD 435

Each kit contained five spin columns that produced 2 ml of biotinylated antibody each. Thus, each kit produced 10ml of stock concentration biotinylated antibody. The antibody working dilution (1:600) produced 6 000 ml of biotinylated antibody. Each slide (containing one touch impression) required approximately 0.05 ml of biotinylated antibody.

$$\frac{\text{6 000 ml biotinylated antibody per kit}}{\text{0,05 ml biotinylated antibody per slide}} = \text{120 000 slides per kit}$$

$$\frac{\text{USD 435 per kit}}{\text{120 000 slides per kit}} = \textbf{\$0,0036 per sample}$$

C2) 10% Neutral buffered formalin (20L) @ USD120

$$\frac{\text{20 000 ml of 10\% Neutral buffered formalin}}{\text{140 ml of Neutral buffered formalin}} = \text{142 runs per bottle}$$

$$\frac{\text{USD 120 for 20L of 10\% Neutral buffered formalin}}{\text{142 runs per bottle of 10\% Neutral buffered formalin}} = \text{\$0,85 per 140 ml of 10\% Neutral buffered formalin}$$

**1 Sample per run – Low throughput facility**	**2 Samples per run – Medium throughput facility**	**5 Samples per run – High throughput facility**
USD 0,85 per 140 ml	USD 0,85 per 140 ml	USD 0,85 per 140 ml
1 slide per 140 ml<br>=<br>**$0,85 per sample**	2 slides per 140 ml<br>=<br>**$0,43 per sample**	5 slides per 140 ml<br>=<br>**$0,17 per sample**

C3) 30% Hydrogen peroxide (2,5L) @ USD 11

The standard operating procedure for the dRIT diagnostic assay required the use of 3% Hydrogen peroxide.

25 000 ml 3% Hydrogen Peroxide / 140ml Hydrogen Peroxide per run = 178 runs per bottle

USD 11 per bottle 3% Hydrogen Peroxide / 178 runs per bottle = $0,06 per 140ml of 3% Hydrogen Peroxide

1 Sample per run – Low throughput facility	2 Samples per run – Medium throughput facility	5 Samples per run – High throughput facility
USD 0,06 per 140 ml	USD 0,06 per 140 ml	USD 0,06 per 140 ml
1 slide per 140 ml<br>=<br>**$0,06 per sample**	2 slides per 140 ml<br>=<br>**$0,03 per sample**	5 slides per 140 ml<br>=<br>**$0,012 per sample**

C4) Streptavidin-peroxidase (100ml, Ready-to-use) @ USD 256

100 ml per bottle of Streptavidin peroxidase / 0,05 ml per slide of Streptavidin peroxidase = 2 000 slides per bottle

256 USD per bottle / 2000 slides per bottle = **$0,128 per sample**

C5) AEC Chromogen kit @ USD 167

150 ml of AEC Chromogen staining kit / 0,05 ml AEC chromogen per slide = 3 000 slides per bottle

USD 167 per AEC Chromogen staining kit / 3000 slides per AEC Chromogen staining kit = **$0,056 per sample**

C6) Gills #2 Haematoxylin (1L) @ USD 57

According to the standard operating procedure of the dRIT diagnostic assay, the Gills #2 Haematoxylin solution is diluted 1:2 and made once a week (each week consisted of five working days). The volume of the full submersion chamber was 140 ml and 45 ml Gills #2 solution was required to ensure sufficient dilution.

$$\frac{\text{2 000 ml per bottle of Gills\#2 solution}}{\text{45 ml Gills\#2 solution per week}} = \text{44 weeks per bottle}$$

$$\frac{\text{USD 57 per bottle of Gills\#2 solution}}{\text{44 weeks per bottle of Gills\#2 solution}} = \text{\$1,30 per week}$$

1 Sample per run – Low throughput facility	2 Samples per run – Medium throughput facility	5 Samples per run – High throughput facility
USD 1,30 per week	USD 1,30 per week	USD 1,30 per week
1 slide per week<br>=<br>**$1,30 per sample**	10 slides per week<br>=<br>**$0,13 per sample**	20 slides per week<br>=<br>**$0,065 per sample**

C7) 1L (x10) PBS @ USD 27

The standard operating procedure for the dRIT diagnostic assay required the use of 1xPBS. The three individual PBS wash steps with each wash step relying on 140 ml. The final volume of 1xPBS per diagnostic run was thus 420 ml.

$$\frac{\text{10 000 ml per bottle 1xPBS}}{\text{420 ml per run}} = \text{23 runs per bottle}$$

$$\frac{\text{USD 27 per 20 L of 1xPBS}}{\text{23 runs per bottle}} = \text{USD 1,17 per 420 ml of 1xPBS}$$

1 Sample per run – Low throughput facility	2 Samples per run – Medium throughput facility	5 Samples per run – High throughput facility
USD 1,17 per 420 ml	USD 1,17 per 420 ml	USD 1,17 per 420 ml
1 slide per run<br>=<br>**$1,17 per sample**	2 slides per run<br>=<br>**$0,60 per sample**	5 slides per run<br>=<br>**$0,23 per sample**

C8) Tween80 (500ml) @ USD 14

According to the standard operating procedure of the dRIT diagnostic assay, for every 990 ml of PBS, 10 ml of Tween80 had to be added. Thus, for every 420 ml of PBS, 4,2 ml of Tween 80 was required.

$$\frac{\text{500 ml per bottle of Tween80}}{\text{4,2 ml Tween80 per run}} = \text{119 runs per bottle of Tween80}$$

$$\frac{\text{USD 14 per bottle of Tween80}}{\text{119 runs per bottle of Tween80}} = \text{USD 0,12 per run}$$

1 Sample per run – Low throughput facility	2 Samples per run – Medium throughput facility	5 Samples per run – High throughput facility
USD 0,12 per 140 ml	USD 0,12 per 140 ml	USD 0,12 per 140 ml
1 slide per 140 ml<br>=<br>**$0,12 per sample**	2 slides per 140 ml<br>=<br>**$0,06 per sample**	5 slides per 140 ml<br>=<br>**$0,024 per sample**

C9) Microscope slides (pack of 50) @ USD 3

$$\frac{\text{USD 3 per pack}}{\text{50 slides per pack}} = \textbf{\$0,06 per sample}$$

C10) Cover slips (pack of 100) @ USD4,20

$$\frac{\text{USD 4,20 per pack}}{\text{100 cover slips per pack}} = \textbf{\$0,04 per sample}$$

Average price of reagents per diagnostic run of the dRIT diagnostic assay	
D1)	Nitrile gloves (box of 100) @ USD 6,50 per box<br>Two pairs of gloves (n = 4) required per diagnostic run according to the double glove policy

$$\frac{\text{100 gloves per box}}{\text{USD 6.5 per box}} = \$0,0065 \text{ per glove}$$

$0,0065 x 4 = **$0,0260** per run (regardless of sample number)

D2)	Pipettor tips (1 – 200 ul) @ USD 10 for 96 tips<br>2 tips required per sample (1 to add the AEC chromogen and 1 to add mounting media to the impression

$$\frac{\text{USD 10 per tray}}{\text{96 tips per tray}} = \mathbf{\$0,10 \text{ per tip}}$$

1 Sample per run – Low throughput	2 Samples per run – Medium	5 Samples per run – High
$0,1042 x 2	$0,1042 x 4	$0,1042 x 10
=	=	=
**$0,21 per run**	**$0,42 per run**	**$1,04 per run**
