# Supplementary material for: Economic and feasibility comparison of the dRIT and DFA for decentralized rabies diagnosis in resource-limited settings: The use of Nigerian dog meat markets as a case study
Source: PLoS Negl Trop Dis. 2020 Feb 28;14(2):e0008088. doi: 10.1371/journal.pntd.0008088 (PMC7065817; doi:10.1371/journal.pntd.0008088)
Supplement: S2 File — (DOCX) [file pntd.0008088.s002.docx]

**S2. Neuronal tissue sample cohort from Nigeria depicting the estimated viral RNA copy numbers as determined using a quantitative real-time polymerase chain reaction assay.**

| **Number** | **Sample number** | **qRT-PCR**  **(South Africa)** | **Estimated RNA copy number** |
| --- | --- | --- | --- |
| 1 | 49 | Positive | [3.05E9] |
| 2 | 50 | Positive | [1.51E9] |
| 3 | 65 | Positive | [9.78E8] |
| 4 | 95 | Positive | [2.20E8] |
| 5 | 140 | Positive | [3.72E8] |
| 6 | 142 | Positive | [9.11E8] |
| 7 | 176 | Positive | [4.28E8] |
| 8 | 185 | Positive | [1.38E9] |
| 9 | 249 | Positive | [5.76E8] |
| 10 | 251 | Positive | [1.83E9] |
| 11 | 261 | Positive | [1.35E9] |
| 12 | 262 | Positive | [2.56E9] |
| 13 | 263 | Positive | [2.81E8] |
| 14 | 264 | Positive | [1.52E8] |
| **15*** | **265** | **Positive** | **[9.12E3]** |
| 16 | 266 | Positive | [2.47E9] |
| 17 | 267 | Positive | [2.26E9] |
| 18 | 268 | Positive | [6.15E9] |
| 19 | 269 | Positive | [8.57E9] |
| 20 | 270 | Positive | [5.80E9] |
| **21*** | **271** | **Positive** | **[1.67E4]** |
| 22 | 272 | Positive | [1.19E9] |
| **23*** | **273** | **Positive** | **[>1.04E3]** |
| 24 | 274 | Positive | [4.38E8] |
| 25^#^ | 275 | Negative | - |
| 26 | 276 | Positive | [5.12E9] |
| **27*** | **277** | **Positive** | **[6.22E4]** |
| **28*** | **278** | **Positive** | **[8.69E4]** |
| “*” indicates that hemi-nested PCR amplicons were also obtained, “#” indicates that the hemi-nested PCR was applied but that no amplicons were obtained.  qRT-PCR: quantitative real-time polymerase chain reaction | | | |
